# Management of Early Stage, High-Risk Endometrial Carcinoma: Preoperative and Surgical Considerations

**DOI:** 10.1155/2013/757249

**Published:** 2013-06-25

**Authors:** Sareena Singh, Shandhini Raidoo, Gaetan Pettigrew, Robert DeBernardo

**Affiliations:** Division of Gynecologic Oncology, Department of Obstetrics and Gynecology, University Hospitals Case Medical Center, Cleveland, OH 44106, USA

## Abstract

Endometrial cancer is the most common gynecologic malignancy in the developed world. Most cases are diagnosed at an early stage and have low-grade histology, portending an overall excellent prognosis. There exists a subgroup of patients with early, high-risk disease, whose management remains controversial, as current data is clouded by inclusion of early stage tumors with different high-risk features for recurrence, unstandardized protocols for surgical staging, and an evolving staging system by which we are grouping these patients. Here, we present preoperative and intraoperative considerations that should be taken into account when planning surgical management for this population of patients.

## 1. Introduction

Endometrial cancer is the most common gynecologic malignancy in the developed world. In 2013, it is estimated that over 49,000 new cases will be diagnosed and 4,000 deaths will be reported in the United States [1]. The overall prognosis is excellent for most patients diagnosed with early stage and low-grade disease. Recently, there has been a movement away from comprehensive surgical staging for women with endometrial cancer. This is driven, in part, by the lack of apparent benefit seen in a number of studies along with the presumed morbidity ascribed to lymphadenectomy. There are, however, well-established risk factors for patients with early stage disease which may confer a poorer survival advantage, including increasing age, grade 2-3 histology, positive lymphovascular space involvement, and outer 1/3 myometrial involvement [2]. This high-intermediate risk group of patients is defined by (1) any age with 3 of the above risk factors, (2) age ≥ 50 years with 2 of the above risk factors, and (3) age ≥ 70 years with at least one of the above risk factors [3]. The impact of surgical staging in the cohort of women with early stage disease with high to intermediate risk factors has not been investigated. Instead, these patients have been “lumped together” with women with low-risk disease, potentially obscuring any observable benefit. Further, in this subset of patients, adjuvant therapy may impact progression free and overall survival. The management of this subset of women is the focus of this paper.

Management of patients with high to intermediate risk endometrial cancer is controversial. This is not surprising as the published literature that we may use to inform intraoperative and adjuvant treatment decisions is difficult to interpret. Studies sometimes include patients that have undergone comprehensive surgical staging that are incompletely staged or that have clinically stage I disease (no lymphadenectomy). Further complicating matters, the International Federation of Gynecology and Obstetrics (FIGO) staging system has changed, thereby muddying the interpretation of results of previous published studies. Finally, the wide-spread adoption of laparoscopy and robotics in the management of this disease has modified the short-term morbidity associated with surgical management. There is a paucity of data, however, on long-term cancer-related outcomes in these patients with early stage, high-intermediate risk disease.

It is time to reassess what we know, or think we know, about endometrial cancer. We plan to critically re-review the pertinent data to help address the following questions in this high to intermediate risk cohort: to stage or not to stage? How do we triage whom to stage? Which surgical approach is most appropriate—open, laparoscopic, or robotic? How can we use intraoperative assessments to guide surgical decision making? What do we know about the morbidity of lymphadenectomy? What should we do with unstaged, high/intermediate risk patients? How does this differ if they are comprehensively staged? What is the role of adjuvant radiation and chemotherapy?

## 2. Figo Staging of Endometrial Cancer

Endometrial cancer was clinically staged until 1988, when a surgical staging system was adopted by the International Federation of Gynecology and Obstetrics. Endometrial cancer staging includes a total hysterectomy, bilateral salpingo-oophorectomy (BSO), and systematic pelvic and para-aortic lymphadenectomy. The 1988 FIGO staging system is shown in Table 1. In 2009, the FIGO staging guidelines were modified as depicted in Table 2. The changes in the updated staging system reflected newly available data on prognostic factors that affected patient the outcomes. Previous stages IA (noninvasive) and IB (<50% myometrial invasion) were combined into stage IA. This was based on similar five-year survival rates observed in these two cohorts [4]. In the earlier FIGO staging system, cervical glandular involvement (IIA) was distinguished from cervical stromal involvement (IIB). Data has subsequently demonstrated that cervical glandular involvement does not adversely affect the outcome, which is reflected by removing this as inclusion criteria for stage II patients in the current system. Nodal involvement is now stratified into stage IIIC1 (positive pelvic lymph nodes) and IIIC2 (positive para-aortic lymph nodes with or without positive pelvic lymph nodes) based on data suggesting a worse prognosis with positive para-aortic lymph nodes [5, 6]. Peritoneal cytology was a controversial part of the prior staging system but no longer affects the stage of disease.

The change in the staging of endometrial cancer is paramount not only in guiding treatment decisions but also impacts how we interpret the literature. Studies published before 2009 include patients that were stratified differently than our current practice. Recognizing this, we need to be aware that patients in these studies may be at a significantly lower risk of recurrence, such as the “old IA” patient compared to “today's IA” patient that may have up to 50% myometrial invasion. In reality, depth of invasion is not a discrete variable, as the staging system artificially creates it, but instead is a continuous variable with risk increasing with each additional millimeter of invasion. We should also keep in mind that, prior to 2009, patients with stage II disease included the cohort of relatively low-risk patients with cervical glandular involvement.

## 3. Preoperative and Intraoperative Evaluation

The best approach to identifying the high-intermediate risk women pre-or intraoperatively has not been determined and often is surgeon dependent. An argument can be made to perform surgical staging on all women with endometrial cancer regardless of the grade so as to identify all women with advanced disease. Others recommend staging only a selected group of patients to avoid morbidity, citing the apparent lack of survival benefit from comprehensive surgical staging as a rationale for this approach. The decision of who to surgically stage, however, is probably best determined deliberately. Understanding several key factors is critical to making an informed decision regarding the proper surgical procedure: assessing grade, depth of myometrial invasion, tumor size, and lymph node evaluation.

It has long been recognized that the tumor grade is an important determinant of lymph node metastases. There are many ways in which tumor grade can be assessed preoperatively, including office endometrial biopsy and dilation and curettage (D&C). The accuracy of each of these methods differs. A retrospective study by Larson et al. examined the use of office pipelle sampling compared with D&C to determine the histologic grade in patients with known endometrial cancer [7]. Pipelle biopsy correctly identified the hysterectomy tumor grade in 76 out of 131 patients (58%), and D&C correctly identified tumor grade in 40 out of 52 patients (77%). Office biopsy was inaccurate 42% of the time, and notably 26% of these discrepant cases were upgraded on final pathology. Only 10% of inaccurate D&C specimens were upgraded to a higher grade based on final pathology. When using the grade to determine the need for lymphadenectomy, the method of sampling must be considered. Patients having undergone a D&C are half as likely to be found to have a higher grade malignancy on final pathology. Frozen section analysis of hysterectomy specimens can also be used to determine the tumor grade, with a reasonably high accuracy [8, 9]. Reported rates of concordance between intraoperative frozen section and permanent section for assessing tumor grade are approximately 90%.

Assessing the depth of myometrial invasion is another important aspect of determining the correct surgical procedure for patients with endometrial cancer. Much like grade, depth of invasion can be determined preoperatively, using ultrasound or MRI, but also intraoperatively, with gross visual inspection or frozen section analysis. The diagnostic accuracy for correctly assessing depth of invasion with MRI ranges from 68% to 85% [10, 11] and for ultrasound ranges from 68% to 77% [10, 12]. The routine use of this technology is limited in clinical practice as most centers lack a radiologist with the sufficient experience to provide accurate interpretation. Many studies have examined intraoperative assessment of depth of myometrial invasion at the time of hysterectomy using gross visual inspection by the surgeon and frozen section assessment. A recent meta-analysis of 16 studies looking at intraoperative gross examination of hysterectomy specimens reported an accuracy of 87% in determining depth of myometrial invasion [13]. Accuracy of frozen section analysis to determine depth of invasion has been reported as high as 85–93% [8, 9].

Preoperative assessment of lymph node involvement using various radiologic imaging modalities and intraoperative assessment of lymph node involvement by palpation have been proposed by some as useful techniques to assess for metastasis. Unfortunately, for patients undergoing surgery via robotic or laparoscopic approaches, retroperitoneal palpation is impossible. Moreover, the data does not support this approach with regard to diagnostic accuracy [14]. Even when performed by experienced gynecologic oncologists, palpation of the retroperitoneum to identify suspicious lymph nodes can lead to up to 1/3 of positive lymph nodes being misdiagnosed as clinically negative. Using palpation to assess whether lymphadenectomy should be performed is less accurate than flipping of a coin and therefore cannot be relied upon to guide surgical decision making. The accuracy of MRI and PET-CT for detection of lymph nodes metastases has been reported up to 90%; however these imaging modalities are not sensitive enough to completely replace surgical staging [15]. Further, much of this data was obtained from patients at higher risk of nodal metastasis than the high to intermediate risk patients cohort making actual sensitivity and specificity lower than predicted.

Current techniques available for preoperative and intraoperative assessment of tumor characteristics for the purposes of guiding surgical decision making have been shown to have a high degree of accuracy. If preoperative sampling is performed via pipelle endometrial biopsy rather than D&C, there is a higher chance that the tumor will be upgraded on final pathology. Frozen section analysis for grade determination should be strongly considered if this is the case. Gross visual inspection for depth of invasion is fairly reliable. Unfortunately, there are no current methods to determine the presence or absence of lymphovascular space involvement intraoperatively. It should also be recognized that the accuracy of gross inspection falls with increasing the grade of tumor; hence the threshold to perform systemic lymphadenectomy should be lower in this setting.

## 4. Role of Lymphadenectomy

The role of lymphadenectomy in the surgical management of women with early stage endometrial cancer has not been standardized and has led to widely differing practice patterns in the United States. There are still 2 major questions remaining with regards to lymphadenectomy in patients with endometrial cancer (1) Which patients with clinical early stage disease should undergo lymphadenectomy? (2) How extensive of a lymph node dissection should be performed? Two large trials were conducted to help answer these questions.

The Adjuvant External Beam Radiotherapy in the Treatment of Endometrial Cancer (ASTEC) Trial was a multicenter prospective randomized trial conducted from 1998 to 2005 in Europe [16]. Fourteen-hundred and eight subjects were randomized to standard surgery (hysterectomy, BSO, peritoneal washings, and palpation of para-aortic nodes; *n* = 704) or standard surgery plus bilateral pelvic lymphadenectomy (*n* = 704). The decision to perform para-aortic lymphadenectomy was left to the discretion of the surgeon. The primary outcome of this study was overall survival. The median time of followup was 37 months. Although patients in this study were randomized, baseline characteristics differed significantly between the 2 groups, with more aggressive histologic subtypes and tumors with greater depths of invasion found in the lymphadenectomy arm. These differences were corrected statistically. The hazard ratio for overall survival was 1.04 (95% CI 0.74–1.45; *P* = 0.83) and for recurrence free survival was 1.25 (95% CI 0.93–1.66; *P* = 0.14). Study authors concluded that there were no overall or recurrence-free survival benefits for performing lymphadenectomy and that pelvic lymphadenectomy could not be routinely recommended. Several limitations, however, have been identified with this study, including inadequate pelvic lymph node sampling (median number of resected lymph nodes was 9), inclusion of a large number of patients with low-risk features that may not have ever benefitted from lymphadenectomy, and leaving the decision to perform para-aortic node dissection up to the physician's preference.

The CONSORT Trial was another Phase III randomized trial that was conducted in Italy to help discern the clinical impact of lymphadenectomy [17]. This study was conducted from 1996 to 2006 and randomized 514 patients to undergo pelvic lymphadenectomy at the time of hysterectomy and BSO (*n* = 264) or no lymphadenectomy. Similar to the ASTEC trial, performing para-aortic lymph node dissection was left to the discretion of the surgeon. Median followup was 49 months. Disease free survival did not differ significantly between the lymphadenectomy group and the no lymphadenectomy group (81% versus 81.7%). Overall survival also did not differ significantly (85.9% versus 90%). A major criticism of this trial is that the indications for and the extent of lymphadenectomy were not clearly defined.

Despite these 2 large prospective trials having been conducted, we still do not have a clear answer about the benefit of lymphadenectomy in early stage patients. Furthermore, it also remains unclear how aggressive of a lymphadenectomy should be performed. Researchers at Mayo designed a prospective trial to help assess lymphatic dissemination patterns [18]. This study showed that in patients with positive nodal metastases, 51% had both pelvic and para-aortic nodes that were positive and that 16% had only para-aortic nodes that were positive. Of the subjects that had positive para-aortic nodes, 77% had nodes above the level of the inferior mesenteric artery that were positive, thereby creating an argument for extending aortic lymphadenectomy up to the renal vessels when performed. The same group also conducted a study to assess survival, morbidity, and cost associated with performing lymph node dissection in patients at low risk for nodal metastases (primary tumor diameter ≤ 2 cm, depth of invasion ≤ 50%, and grade 1-2 histology) [19]. The authors of this study concluded that the incorporation of lymphadenectomy in the surgical management of patients that have low-risk disease (as defined by the Mayo criteria) increases morbidity and cost and does not provide any observable benefit. They suggest that the standard of care for these patients should be hysterectomy and BSO only. This data, however, should be interpreted with some caution since it may lack external validity given that not all hospital systems have the same access to and quality of frozen section analysis.

After critically reviewing these 2 trials, there is still no clear evidence to promote complete surgical staging in women with low-risk stage I endometrial cancer. However, there were not enough early stage high-intermediate risk patients included in these trials to be able to draw separate conclusions about this subset of patients. Complete surgical staging of women with high-intermediate risk features should still be strongly considered.

## 5. Minimally Invasive Surgery

Historically, the surgical approach for the management of all stages of endometrial cancer has been via laparotomy. However, with the increasing incorporation of minimally invasive surgical techniques into gynecology, the use of a laparoscopic approach (both standard laparoscopic and robotic platforms) for treatment of early stage endometrial cancer has become more commonplace. The already established general benefits of minimally invasive surgery, such as decreased blood loss, shorter recovery time, and fewer postoperative complications, become especially attractive when dealing with a population of endometrial cancer patients, who are typically obese with multiple medical comorbidities.

A randomized, phase III trial conducted by the Gynecologic Oncology Group (LAP2) was designed with the objective of comparing laparoscopy versus laparotomy for the comprehensive surgical staging of women with clinical stage I to IIA uterine cancers [20]. This trial enrolled patients from 1996 to 2005 and randomized subjects in a 2 : 1 ratio to a laparoscopic versus open approach to hysterectomy, bilateral salpingo-oophorectomy, and pelvic and para-aortic lymph node sampling. This study was designed as a noninferiority trial, with the primary study endpoint of recurrence-free interval. A total of 2,616 patients were enrolled and randomly assigned, with 920 patients undergoing laparotomy and 1,696 undergoing surgery via laparoscopy.

Preliminary study outcomes from LAP2 were published in 2009 [20]. This first report demonstrated that surgery via a laparoscopic approach resulted in shorter hospital stays (with 52% of patients in the laparoscopy group requiring a hospital stay >2 days versus 94% in the laparotomy group, *P* < 0.0001), fewer perioperative complications (14% versus 21%, *P* < 0.001), longer operative times (204 versus 130 minutes, *P* < 0.001), and similar rates of intraoperative complications (10% versus 8%, *P* = 0.106). Twenty-six percent of patients in the laparoscopy arm, however, required conversion to an open approach. Risk factors for conversion with the strongest correlation included increased body-mass index, older age, and presence of metastatic disease. Removal of pelvic and para-aortic lymph nodes differed significantly between the 2 groups—96% of laparotomy patients and 92% of laparoscopy patients underwent lymph node dissection (*P* < 0.0001). An equal number of patients in both groups (17%), however, were found to have metastatic disease. These results allowed us to conclude that the laparoscopic approach to staging was safe and did not compromise the ability to perform an adequate staging procedure.

Survival data from LAP2 were published in 2012 [21]. During the median follow-up time of 59 months, 309 recurrences (210 in the laparoscopy group and 99 in the laparotomy group) were detected and 350 deaths (229 in the laparoscopy group and 121 in the laparotomy group) occurred. The hazard ratio for laparoscopy was reported as 1.14 (95% CI 0.92–1.46), which did not meet the protocol-specific criteria for establishing the noninferiority. Also, the 3-year estimated cumulative incidence of recurrence in the laparoscopy arm was 11.39% compared to 10.24% in the laparotomy arm. The difference between recurrence at the 3-year mark was 1.14% (95% CI—1.278 to 3.996), which also did not meet the study's design to demonstrate noninferiority. The estimated 5-year recurrence rates were 13.68% for laparoscopy subjects and 11.61% for laparotomy patients. No differences in sites of cancer recurrence were observed between the 2 groups. The major disadvantages seen when interpreting the results of the LAP2 study lie in the fact that this study was designed as a noninferiority trial and that the predetermined threshold for noninferiority was not reached, thereby making the results regarding survival inconclusive. Two major factors that contributed to the results being inconclusive have been identified [22]. First, the endpoints of recurrence and survival were not originally designated to be the endpoints in the trial's design, which led to the issue of almost a quarter of subjects being followed for less than 3 years. Second, study design was based on a projected recurrence rate of 15%, which, although a large number of subjects were included, resulted in a relatively underpowered study. These important design flaws result in our inability to confidently exclude the possibility of a small difference between the 2 groups actually existing. Any difference, even if small, would be compounded in the high-intermediate risk group of patients. Finally, LAP2 was not powered to adequately detect a difference between Type 1 and Type 2 endometrial cancers, with regards to patterns of cancer recurrence.

There have been a number of other studies that have also addressed the issue of incorporating minimally invasive approaches into the surgical management of early stage endometrial cancer. A multi-institutional, randomized trial was conducted in the Netherlands from 2007 to 2009, which compared total abdominal hysterectomy versus total laparoscopic hysterectomy for the management of patients with stage I endometrial cancer or complex atypical hyperplasia [23]. The primary outcome was the rate of major complications. No differences between surgical approaches were observed with regards to the rate of major complications, but patients in the laparoscopy group benefited from less estimated blood loss, shorter hospital stays, and overall faster recoveries. Similar findings, that morbidity is lower with minimally invasive techniques and that survival and recurrence rates do not differ greatly, have also been corroborated retrospectively [24].

Robotic surgery has now also become a feasible option for use in the surgical management of early stage endometrial cancers. Multiple retrospective cohort studies have looked at the outcomes and costs of performing staging procedures for endometrial cancer with robotic assistance versus standard laparoscopy and laparotomy [25–27]. Advantages of a robotic platform as compared to standard laparoscopy include use of 3-dimensional imaging, instruments with wide ranges of motion, and a faster learning curve [28]. These studies conclude that surgery via robotic platform results in adequate surgical staging and decreased morbidity. However, costs associated with the use of robotic assistance, which include cost of training and equipment maintenance in addition to the initial investment, remain very high. On a long term, this cost may be negated by decrease in postoperative complications, hospital stays, and readmissions but this has yet to be determined. Long-term oncologic outcomes of patients undergoing robotically assisted staging have not yet been established.

Many factors must be considered when determining the ideal surgical approach for the optimal outcome and the patient benefit. There are advantages and disadvantages to all possible surgical approaches, which makes the decision regarding which approach to pursue a challenging one. At present there will likely always be a place for both laparotomy and laparoscopy. Given the benefits of robotics it would seem unwise for it not to have a place in the armamentarium against early stage endometrial cancer. As we continue to make advances in surgical techniques and technologies, new approaches to surgical management may be implemented and adopted. Continued studies, especially those that include patients with high-intermediate risk features, are necessary to determine the best approach in terms of outcome and cost.

## 6. Conclusions

Management of women with high-intermediate risk, early stage endometrial cancer remains controversial, and practice patterns vary widely among gynecologic oncologists. While a large amount of time and resources have been invested into the study of women with early stage endometrial cancer, differences in outcomes are difficult to establish since these patients tend to have an overall excellent prognosis. Current data is clouded by inclusion of early stage tumors with different high-risk features for recurrence, unstandardized protocols for surgical staging and an evolving staging system by which we are grouping these patients. Given that a high-intermediate risk population of patients with early stage exists which could benefit from extended surgical staging and adjuvant treatments, we need to invest more into characterizing recurrence patterns and treatment effects in order to adequately treat these patients.

## 7. Future Directions

Preoperative and intraoperative classifications of tumor characteristics through routine and frozen histopathologic assessments can provide us only with information regarding histology, grade, size, and depth of invasion. This information, with the addition of presence or absence of lymphovascular space involvement, can be confirmed by final histopathologic analysis. However, there has now been increased interest in classifying tumor biology based on data obtained from integrated genomic and proteomic analyses of endometrial cancers [29]. Using this technology, more direct insights into tumor biology via classification into types of tumors with similar behaviors can be discerned, allowing for more tailored adjuvant therapies. In this paper, the authors performed multiplatform analyses of over 370 endometrial carcinomas, including low-grade endometrioid, high-grade endometiriod, and serous carcinomas. They were able to identify 4 new groups of tumor types, based on integrated genomic data: (1) *POLE *ultramutated, (2) microsatelite instability hypermutated, (3) copy-number low, and (4) copy-number high. It has been shown that high grade endometrioid and high grade serous carcinomas can at times be difficult to subtype correctly, with not insignificant rates of intraobserver concordance among pathologists [30, 31].

Correct classification is of paramount importance, as often Type I endometrial cancers are treated with adjuvant radiation and Type II endometrial cancers are treated with adjuvant chemotherapy-based treatments. As an example, approximately 1/4 of tumors that were classified as high-grade endometrioid on pathologic review actually had similar phenotypes as uterine serous carcinomas when genomic-based analyses were performed. Incorporation of genomic analyses for the purposes of more specific tumor classification into clinical practice may provide us with a way to more appropriately tailor post-operative adjuvant treatments.

## Figures and Tables

**Table 1:** 1998 FIGO staging system for endometrial cancer.

Stage	Anatomic involvement
Stage I	Tumor confined to the uterine corpus
IA	No myometrial invasion
IB	<50% myometrial invasion
IC	≥50% myometrial invasion
Stage II	Cervical involvement
IIA	Endocervical glandular involvement
IIB	Cervical stromal invasion
Stage III	
IIIA	Positive peritoneal cytology and/or tumor invasion into uterine serosa and/or adnexal involvement
IIIB	Vaginal involvement
IIIC	Metastases to pelvic and/or pelvic lymph nodes
Stage IV	
IVA	Bladder and/or bowel involvement
IVB	Distant metastases, including abdominal disease and/or inguinal lymph node involvement

**Table 2:** 2009 FIGO staging system for endometrial cancer.

Stage	Anatomic involvement
Stage I	Tumor confined to the uterine corpus
IA	No or <50% myometrial invasion
IB	≥50% myometrial invasion
Stage II	Cervical stromal involvement
Stage III	Local and/or regional tumor spread
IIIA	Tumor invasion into uterine serosa and/or adnexal involvement
IIIB	Vaginal and/or parametrial involvement
IIIC	Metastases to lymph nodes
IIIC1	Positive pelvic lymph nodes
IIIC2	Positive para-aortic lymph nodes
Stage IV	
IVA	Bladder and/or bowel involvement
IVB	Distant metastases, including abdominal disease and/or inguinal lymph node involvement

## References

[1] (2013). *Cancer Facts & Figures—2013*.

[2] Creutzberg CL, Van Putten WLJ, Koper PC (2003). Survival after relapse in patients with endometrial cancer: results from a randomized trial. *Gynecologic Oncology*.

[3] Keys HM, Roberts JA, Brunetto VL (2004). A phase III trial of surgery with or without adjunctive external pelvic radiation therapy in intermediate risk endometrial adenocarcinoma: a gynecologic oncology group study. *Gynecologic Oncology*.

[4] Creasman W (2009). Revised FIGO staging for carcinoma of the endometrium. *International Journal of Gynecology and Obstetrics*.

[5] Morrow CP, Bundy BN, Kurman RJ (1991). Relationship between surgical-pathological risk factors and outcome in clinical stage I and II carcinoma of the endometrium: a gynecologic oncology group study. *Gynecologic Oncology*.

[6] Hoekstra AV, Kim RJ, Small W (2009). FIGO stage IIIC endometrial carcinoma: prognostic factors and outcomes. *Gynecologic Oncology*.

[7] Larson DM, Johnson KK, Broste SK, Krawisz BR, Kresl JJ (1995). Comparison of D and C and office endometrial biopsy in predicting final histopathologic grade in endometrial cancer. *Obstetrics and Gynecology*.

[8] Turan T, Oguz E, Unlubilgin E (2013). Accuracy of frozen-section examination for myometrial invasion and grade in endometrial cancer. *European Journal of Obstetrics & Gynecology and Reproductive Biology*.

[9] Çetin Ç, Özdemir S, Esen H, Balc O, Yilmaz O (2010). The clinical value of preoperative and intraoperative assessments in the management of endometrial cancer. *International Journal of Gynecological Cancer*.

[10] Yamashita Y, Mizutani H, Torashima M (1993). Assessment of myometrial invasion by endometrial carcinoma: transvaginal sonography vs contrast-enhanced MR imaging. *American Journal of Roentgenology*.

[11] Manfredi R, Mirk P, Maresca G (2004). Local-regional staging of endometrial carcinoma: role of MR imaging in surgical planning. *Radiology*.

[12] Akbayir O, Corbacioglu A, Numanoglu C (2011). Preoperative assessment of myometrial and cervical invasion in endometrial carcinoma by transvaginal ultrasound. *Gynecologic Oncology*.

[13] Mavromatis ID, Antonopoulos CN, Matsoukis IL (2012). Validity of intraoperative gross examination of myometrial invasion in patients with endometrial cancer: a meta-analysis. *Acta Obstetricia et Gynecologica Scandinavica*.

[14] Arango HA, Hoffman MS, Roberts WS, Decesare SL, Fiorica JV, Drake J (2000). Accuracy of lymph node palpation to determine need for lymphadenectomy in gynecologic malignancies. *Obstetrics and Gynecology*.

[15] Antonsen SL, Jensen LN, Loft A (2013). MRI, PET/CT and ultrasound in the preoperative staging of endometrial cancer—a multicenter prospective comparative study. *Gynecologic Oncology*.

[16] Kitchener H, Swart AM, Qian Q, Amos C, Parmar MK (2009). Efficacy of systematic pelvic lymphadenectomy in endometrial cancer (MRC ASTEC trial): a randomised study. *The Lancet*.

[17] Panici PB, Basile S, Maneschi F (2008). Systematic pelvic lymphadenectomy vs no lymphadenectomy in early-stage endometrial carcinoma: randomized clinical trial. *Journal of the National Cancer Institute*.

[18] Mariani A, Dowdy SC, Cliby WA (2008). Prospective assessment of lymphatic dissemination in endometrial cancer: a paradigm shift in surgical staging. *Gynecologic Oncology*.

[19] Dowdy SC, Borah BJ, Bakkum-Gamez JN (2012). Prospective assessment of survival, morbidity, and cost associated with lymphadenectomy in low-risk endometrial cancer. *Gynecologic Oncology*.

[20] Walker JL, Piedmonte MR, Spirtos NM (2009). Laparoscopy compared with laparotomy for comprehensive surgical staging of uterine cancer: gynecologic oncology group Study LAP2. *Journal of Clinical Oncology*.

[21] Walker JL, Piedmonte MR, Spirtos NM (2012). Recurrence and survival after random assignment to laparoscopy versus laparotomy for comprehensive surgical staging of uterine cancer: gynecologic oncology group LAP2 study. *Journal of Clinical Oncology*.

[22] Berchuck A, Alvarez Secord A, Havrilesky LJ (2012). Minimally invasive surgery for endometrial cancer: the horse is already out of the barn. *Journal of Clinical Oncology*.

[23] Mourits MJE, Bijen CB, Arts HJ (2010). Safety of laparoscopy versus laparotomy in early-stage endometrial cancer: a randomised trial. *The Lancet Oncology*.

[24] Obermair A, Manolitsas TP, Leung Y, Hammond IG, McCartney AJ (2004). Total laparoscopic hysterectomy for endometrial cancer: patterns of recurrence and survival. *Gynecologic Oncology*.

[25] Seamon LG, Cohn DE, Henretta MS (2009). Minimally invasive comprehensive surgical staging for endometrial cancer: robotics or laparoscopy?. *Gynecologic Oncology*.

[26] Boggess JF, Gehrig PA, Cantrell L (2008). A comparative study of 3 surgical methods for hysterectomy with staging for endometrial cancer: robotic assistance, laparoscopy, laparotomy. *American Journal of Obstetrics and Gynecology*.

[27] Bell MC, Torgerson J, Seshadri-Kreaden U, Suttle AW, Hunt S (2008). Comparison of outcomes and cost for endometrial cancer staging via traditional laparotomy, standard laparoscopy and robotic techniques. *Gynecologic Oncology*.

[28] Ahlering TE, Skarecky D, Lee D, Clayman RV (2003). Successful transfer of open surgical skills to a laparoscopic environment using a robotic interface: initial experience with laparoscopic radical prostatectomy. *Journal of Urology*.

[29] Kandoth C, Schultz N, Cherniack AD (2013). Integrated genomic characterization of endometrial carcinoma. *Nature*.

[30] Clarke BA, Gilks CB (2010). Endometrial carcinoma: controversies in histopathological assessment of grade and tumour cell type. *Journal of Clinical Pathology*.

[31] Gilks CB, Oliva E, Soslow RA (2013). Poor interobserver reproducibility in the diagnosis of high-grade endometrial carcinoma. *The American Journal of Surgical Pathology*.

